# Evaluation of Cattle for Naturally Colonized Shiga Toxin-Producing *Escherichia coli* Requires Combinatorial Strategies

**DOI:** 10.1155/2021/6673202

**Published:** 2021-04-01

**Authors:** Indira T. Kudva, Eben R. Oosthuysen, Bryan Wheeler, Clint A. Loest

**Affiliations:** ^1^Food Safety and Enteric Pathogens Research Unit, National Animal Disease Center, Agricultural Research Service, U.S. Department of Agriculture, Ames, IA, USA; ^2^Department of Animal and Range Sciences, New Mexico State University, Las Cruces, NW, USA

## Abstract

Shiga toxin-producing *Escherichia coli* (STEC) serogroups O157, O26, O103, O111, O121, O145, and O45 are designated as food adulterants by the U.S. Department of Agriculture-Food Safety and Inspection Service. Cattle are the primary reservoir of these human pathogens. In this study, 59 Angus crossbred heifers were tested specifically for these seven STEC serogroups using a combination of standard culture, serological, PCR, and cell cytotoxicity methods to determine if comparable results would be obtained. At the time of fecal sampling, the animals were approximately 2 years old and weighed 1000–1200 lbs. The diet comprised of 37% ground alfalfa hay, 25% ground Sudan hay, and 38% ground corn supplemented with trace minerals and rumensin with ad libitum access to water. Non-O157 STEC were isolated from 25% (15/59) of the animals tested using a combination of EC broth, CHROMagar STEC^TM^, and Rainbow Agar O157. Interestingly, the O157 serogroup was not isolated from any of the animals. Non-O157 STEC isolates were confirmed to be one of the six adulterant serogroups by serology and/or colony PCR in 10/15 animals with the predominant viable, serogroup being O103. PCR using DNA extracted from feces verified most of the colony PCR results but also identified additional virulence and O-antigen genes from samples with no correlating culture results. Shiga toxin- (Stx-) related cytopathic effects on Vero cells with fecal extracts from 55/59 animals could only be associated with the Stx gene profiles obtained by fecal DNA PCR and not culture results. The differences between culture versus fecal DNA PCR and cytotoxicity assay results suggest that the latter two assays reflect the presence of nonviable STEC or infection with STEC not belonging to the seven adulterant serogroups. This study further supports the use of combinatorial culture, serology, and PCR methods to isolate viable STEC that pose a greater food safety threat.

## 1. Introduction

Shiga toxin-producing *Escherichia coli* (STEC) is the third leading cause of foodborne illness after *Campylobacter* and *Salmonella*, implicated in 265,000 illnesses in the US and 2.8 M infections globally [1–3]. A combined economic loss to public health, agriculture, and meat industry estimated at $993 million per year attributed to STEC contamination of foods and human infections prompted the declaration of commonly implicated STEC serogroups (O157, O26, O103, O111, O121, O145, and O45) as food adulterants by the USDA-Food Safety and Inspection Service (FSIS) [2, 4–9]. STEC infections are acquired through the fecal-oral route following ingestion of bacteria-contaminated food or water or after contact with infected animals and humans [10–13]. Following infection, some individuals remain asymptomatic, while others develop watery diarrhea to HC that may exacerbate into often fatal secondary sequelae such as HUS or thrombotic, thrombocytopenic purpura [14]. No specific therapies are available for treating STEC infections in humans. STEC can infect in low doses (∼10 viable bacteria) due to multiple acid tolerance and quorum sensing mechanisms [11, 12, 15, 16]. Virulence factors such as phage-encoded Shiga toxins (Stx) [17], Stx1 and Stx2, plasmid-encoded hemolysis (HlyA) [18], and various adherence factors including intimin, encoded by the *eae* gene on the pathogenicity island locus of enterocyte effacement (LEE), play a significant role in human disease [17].

Cattle are considered the primary STEC reservoirs as most outbreaks are directly or indirectly associated with cattle [19, 20]. Cattle remain asymptomatic due to the absence of the Gb3 receptors for Stx; without uptake of toxin, there is no resulting systemic failure as observed in humans [21–23]. Although STEC can be isolated from various gastrointestinal tract sites, they persist at the rectoanal junction (RAJ) [24, 25]. Average duration of bovine O157 carriage is 30 days, although colonization of up to 1 year has been reported [26–28]. Cattle shed STEC in a seasonal pattern, with increased shedding in warmer months and decreased shedding in winter [20]. Animals shedding greater than 10^4^ CFU/g feces, termed “super-shedders” contribute to herd prevalence of STEC [29, 30]. Postweaned calves and cows tend to be more susceptible to STEC colonization [31–34].

Researchers have used different cultures, immunomagnetic separation (IMS), and PCR methods, solely or in combination, to improve the detection of STEC in field samples although with varied success [35–44]. For instance, when real-time PCR was used to screen 573 bovine fecal samples at slaughter for Stx genes (417/573) and STEC serogroups in the Stx-positive samples, the results did not always correspond with the culture of viable STEC from the same samples using IMS, and when isolated, the colonies did not carry all the targeted virulence genes [45]. Similar comparison of IMS-based culture and conventional and multiplex quantitative PCR, used to analyze 576 bovine fecal samples, demonstrated that these techniques detected all six non-O157 serogroups in samples negative by other methods thereby highlighting the importance of subjecting fecal samples to both culture and PCR for accurate detection of the six non-O157 STEC [46]. Vero cell cytotoxicity assays have been used to predict presence of STEC in feces by correlating cytotoxicity to Stx; however, as with PCR, the results may not always result in the isolation of viable STEC (47–49). To improve the selection and differentiation of the top 6 non-O157 STEC serogroups, a chromogenic agar media was developed that enabled isolation of these serogroups from 114 of the 1897 bovine fecal samples tested [47]. Likewise, in a study evaluating 120 beef cattle, a combination of MacConkey and modified Rainbow® Agar O157 agars increased the recovery frequency of non-O157 STEC strains from animal feces [48].

Based on these reports, in this study, we evaluated a combination of methods to determine the occurrence of O157 and the “Big 6” non-O157 STEC in dairy cows, known to be STEC-susceptible. We compared simple fecal culture techniques followed by serology and colony PCR to direct fecal DNA PCR and Vero cell cytotoxicity assays, in order to ascertain the variability/similarity of results when using these methods to determine the presence of viable O157 and the “Big 6” non-O157 STEC in bovine fecal samples.

## 2. Materials and Methods

### 2.1. Animals and Sampling

Standard husbandry and veterinary care was provided to the animals used in this study, and sampling was carried out as approved by the New Mexico State University (NMSU) Institutional Animal Care and Use Committee. Fecal samples (50 g/animal) were collected by rectal palpation from a total of 59 Angus crossbred heifers, housed at NMSU Clayton Livestock Center in January 2017 (winter), and transported overnight on ice to NADC, Ames, IA, for processing. All samples were collected into sterile Falcon tubes (Thermo Scientific, Rockford, IL) using the appropriate aseptic technique of changing gloves between samples. At the time of sampling, the animals were approximately 2 years old and weighed 1000−1200 lbs with a body condition score of 6 (BCS range 1–9, 9 being extremely fat). All heifers had been artificially inseminated in October 2016 resulting in 52/59 (88%) of the cattle being pregnant as determined by the BioPRYN test that measures pregnancy-specific protein B in serum (Biotracking, Moscow, ID). The animals were comingled and housed in single soil surface pens with partial shade covering. The diet comprised of 37% ground alfalfa hay, 25% ground Sudan hay, and 38% ground corn with ad libitum access to water.

### 2.2. Bacterial Control Strains

Following strains were used as controls to verify culture, latex agglutination, and/or PCR protocols: (i) O157 strain EDL933 (ATCC 43895: *stx*1^+^, *stx*2^+^, *eae*^+^, *hlyA*^+^) (American Type Culture Collection/ATCC, Manassas, VA), (ii) O26:U (NADC 3108: O26^+^, *stx*1^+^, *stx*2^−^, *eae*^+^, *hlyA*^+^) (National Animal Disease Center/NADC, Ames, IA), (iii) O45:U (NADC 3802: O45^+^, *stx*1^+^, *stx*2^−^, *eae*^+^, *hlyA*^+^), (iv) O103:U (NADC 3358: O103^+^, *stx*1^+^, *stx*2^−^, *eae*^+^, *hlyA*^+^), (v) O111:U (NADC 3309: O111^+^, *stx*1^+^, *stx*2^−^, *eae*^+^, *hlyA*^+^), (vi) O121:H19 (ATCC BAA2221: O121^+^, *stx*1^+^, *stx*2^+^, *eae*^+^, *hlyA*^+^), (vii) O121 (ATTC BAA2190: O121^+^, *stx*1^−^, *stx*2^−^, *eae*^−^, *hlyA*^−^), and (viii) O145:U (NADC 3196: O145^+^, *stx*1^−^, *stx*2^−^, *eae*^+^, *hlyA*^+^).

### 2.3. STEC Isolation

#### 2.3.1. O157 Culture

Previously standardized nonenrichment and selective enrichment culture protocols were used to isolate O157 with slight modifications [49–51]. Briefly, per the protocol, 10 g fecal sample was added to 50 ml Trypticase soy broth (BD Bioscience, San Jose, Ca.) supplemented with cefixime (50 *μ*g/liter; U.S. Pharmacopeia, Washington D.C), potassium tellurite (2.5 mg/liter; Sigma-Aldrich Corp., St. Louis, Mo.), and vancomycin (40 mg/liter; Alfa Aesar, Haverhill, Ma.) (TSB-CTV) and mixed well. Serial dilutions of each sample were prepared with sterile saline (0.15 M NaCl) both before and after overnight incubation of the TSB-CTV-fecal suspension at 37°C with aeration. The dilutions prepared before incubation were spread plated onto sorbitol MacConkey agar (BD Biosciences) containing 4-methylumbelliferyl-*β*-d-glucuronide (100 mg/liter; Sigma) (SMAC-MUG) (nonenrichment cultures). SMAC-MUG supplemented with cefixime (50 *μ*g/liter), potassium tellurite (2.5 mg/liter), and vancomycin (40 mg/liter) (SMAC-CTMV) was used to plate the dilutions prepared after overnight incubation (selective-enrichment cultures). Both SMAC-MUG and SMAC-CTMV plates were read after overnight incubation at 37°C, and colonies that did not ferment sorbitol or utilize 4-methylumbelliferyl-*β*-d-glucuronide (nonfluorescent under UV light) were further evaluated to be O157 serologically.

#### 2.3.2. “Big 6” Non-O157 Culture

The six major non-O157 serogroups, O26, O45, O103, O111, O121, and O145, were specifically targeted using a combination of previously reported culture methods [41, 52–55]. As per these protocols, five grams of feces were added to 50 ml *Escherichia coli* (EC) broth (Sigma) and mixed well. Serial dilutions from each EC broth-fecal suspension were prepared with sterile saline (0.15 M NaCl) after overnight incubation at 42°C with aeration. The dilutions were spread plated onto CHROMagar STEC^TM^ supplemented with a 10 ml/L proprietary selective mix provided with the agar (CHROMagar Microbiology, Paris, France). STEC serogroups form mauve-colored colonies on CHROMagar STEC^TM^ (CHROMagar) [41, 52, 54]. Hence, post-overnight incubation at 37^o^C, 15–20 mauve-colored and well-isolated colonies were individually plated on to Rainbow agar O157 (Biolog, Hayward, Ca.). In addition, the fluorescence of the colonies on the CHROMagar STEC^TM^ plates was observed under UV light to differentiate nonfluorescent, mauve O157 from the fluorescent, mauve non-O157 STEC. The Rainbow agar O157 plates were incubated overnight at 37°C and colonies selected based on color for further serological and/or PCR verification of the serogroup as follows: O157, black; O26, O113, O145, and O121:H19, purple; O45, mauve; O103, grey/greyish purple; O111, greyish green [41, 54, 55].

#### 2.3.3. STEC Serology

Latex agglutination tests were used to serologically confirm O157 (*E. coli* O157 latex, Oxoid Diagnostic Reagents, Oxoid Ltd., Hampshire, UK) and the “Big 6” non-O157 (*E.coli* non-O157 Identification Kit, Pro-Lab Diagnostics, Ontario, Canada) serogroups.

### 2.4. Serogroup and Virulence Gene Profiling

#### 2.4.1. Colony Lysates

Colonies (control and fecal isolates) selected for PCR were subcultured from selective plates onto LB plates and used to prepare colony suspensions in sterile distilled water. The suspensions were boiled for 10 min, cooled, and centrifuged, and the lysates are used as template in PCR reactions.

#### 2.4.2. Fecal DNA Extracts

Postincubation, 5 ml of each EC broth-fecal suspension was filtered through a 40*μ* filter, and the filtrate was centrifuged (5000 rpm/10 min/4^o^C) to collect 200–250 mg of fecal material. DNA was extracted from the fecal material using standard instructions provided with the QIAmp DNA stool kit (Qiagen, Germantown, MD). DNA yield and purity were evaluated with the Nanodrop (Life Technologies Corp., Grand Island, NY) and verified by electrophoresis on a 4% agarose gel.

#### 2.4.3. PCR Conditions

Previously described primers [56–58] were used to amplify the *wzx* genes in the O-antigen cluster of non-O157 serogroups and the virulence genes as shown in Table 1. Degenerate primers targeting all variants of the *stx* and *eae* genes were also included (Table 1) [56, 58]. PCR was carried out on the GeneAmp PCR system 9700 thermal cycler (Applied Biosystems) using 10 *μ*l of colony lysate, 200 pmol of each primer, 800 *μ*M deoxynucleoside triphosphates, 1X diluted Ex Taq enzyme buffer, and 2.5 U of TaKaRa Ex Taq DNA polymerase. The hot-start PCR technique was used in combination with a touchdown PCR profile [59] comprising of 20 cycles starting with an annealing temperature of 73°C with touchdown at 53°C at the end of those cycles. Additional amplification segment of 10 cycles was set, using the last annealing temperature of 53°C.

### 2.5. Vero Cell Cytotoxicity Assay

#### 2.5.1. Vero Cell Culture

Vero cells (African Green Monkey Kidney cells, ATCC CCL-81), obtained from the ATCC, Manassas, Va., were grown in Dulbecco's modified Eagle's medium with low glucose, DMEM-LG (Invitrogen, Carlsbad, CA) with additional 10% fetal bovine serum.

#### 2.5.2. Fecal Extract Preparation

Five ml of each EC broth-fecal suspension was filtered through a 40*μ* filter, and the filtrate was centrifuged (1000xg/20 min/4^o^C) to collect debris-free supernatant/fecal extracts.

#### 2.5.3. Assay for Cytotoxicity in the Absence of Antisera

The cytotoxicity assay was conducted as previously described with slight modification [60]. Vero cells were seeded at 10^5^ cells/well in 24-well microtiter plates (Costar, Corning, Ma.) and incubated in the presence of 5% CO_2_ at 37°C for 24 h until confluency was reached. Serial dilutions (1 : 2 to 1 : 64) of the fecal extracts were prepared in DMEM-LG, and 100 *μ*l of each dilution was added per well of the microtiter plates. Plates were incubated for 2 days at 37°C with 5% CO_2_. The cells were microscopically examined for cytotoxicity each day with a final read on the second day. Cytopathic effects (visualized as detached, rounded cells) were numerically scored 1 through 4 corresponding to <25%, 50%, 75%, and >90% cells affected. Control wells with only media were included on each test plate to verify that the cytopathic effects observed were with the fecal extracts or purified toxins alone. Serial dilutions (1 : 2 to 1 : 256) of purified Shiga toxins (Stx-1 and Stx-2; each at a concentration of 50 ng/100 *μ*l) from the NADC stock (NADC, Ames, IA) were tested on the Vero cells separately to validate the procedure.

#### 2.5.4. Cytotoxicity Inhibition in the Presence of Anti-Stx1 or Anti-Stx2 Antisera

The toxin neutralization assay was performed as described previously [61] with slight modification to determine whether the observed CPE was caused by Stx1 and/or Stx2 or other undefined factors. Briefly, microtiter plates with Vero cells and serial dilutions of the fecal extracts were set up as described above. However, in this instance, 100 *μ*l of each fecal extract dilution was mixed with 100 *μ*l polyclonal bovine anti-Stx1 or rabbit anti-Stx2 antisera (NADC stock) and incubated at 37°C/110 rpm followed by overnight incubation at 4°C without shaking. The last dilution at which the fecal extracts produced CPE on Vero cells, in the absence of antisera, was selected for this neutralization assay. A 100 *μ*l sample from each “diluted extract-antisera” mix was added per well of the microtiter plates that were incubated and scored for protection (cells lack CPE) or no protection (cells continue to show CPE). Control wells with only media or fecal extract dilutions were included on each test plate to verify the neutralization effects of the extract-antisera mix, if any. Additionally, purified Shiga toxins (as above), at a dilution of 1 : 256, were mixed with antisera and tested on the Vero cells separately to validate the procedure.

## 3. Results and Discussion

Transportation to processing plants and fasting increase STEC fecal shedding by colonized cattle [26, 62, 63]. STEC on hides are common sources of postharvest (after slaughter) carcass contamination; if STEC colonization of animals goes undetected, these foodborne pathogens could readily spread into packing plants, food processing plants, and consequently enter our food supply [64]. To prevent this farm to fork spread of pathogens, USDA-FSIS instituted the Hazard Analysis Critical Control Point (HACCP) program requiring slaughter facilities to decontaminate at critical carcass processing points [65]. However, efficient STEC control in cattle could enhance the success of the HACCP program [64, 66], and for this, sensitive techniques are needed to detect these foodborne pathogens present in variable concentrations in bovine feces prior to harvesting. Taking into account some of the published studies [35–40, 42–48, 60, 61, 67], we chose a combination of selective culture, serology, conventional colony PCR, direct fecal PCR methods, and cell cytotoxicity assay to detect STEC in 59 bovine fecal samples and determine if these would yield comparable results.

We cultured putative non-O157 STEC from feces of 15/59 (25%) animals tested (Table 2) using EC broth, CHROMagar STEC^TM^, and Rainbow agar O157 plates [41, 52–55] in this study. Utilizing two different selective agar media allowed for a two-tiered differentiation of STEC from background flora. The first step of subculturing fecal samples enriched in the EC broth on to CHROMagar STEC^TM^ plates allowed for identification of fluorescent non-O157 STEC. The second step of selection, in which the fluorescent non-O157 STEC was individually subcultured on to Rainbow agar O157 plates, enabled further differentiation into tentative serogroups based on distinct colony colors that could be easily selected for serological or PCR testing. These non-O157 STEC were confirmed to be one of the “Big 6” serogroups by serology in 10/15 animals (Supplementary Figure 1; Table 2); serogroups O103, O26, and O121 were isolated from 9, 2, and 1 bovine fecal samples, respectively, with O26 and O121 being coisolated with other serogroups (Table 2). Colonies that appeared to be non-O157 STEC, based on the phenotype on the CHROMagar STEC^TM^ and Rainbow agar O157 plates, were also isolated from 5/15 animals, but these could not be assigned to either of the “Big 6” non-O157 STEC based on serology and colony PCR (‘NT'; Table 2). Interestingly, O157 was not isolated from any of the animals tested, despite using a sensitive, selective-enrichment protocol capable of detecting 1 CFU O157/10g feces (Table 2) [50, 51, 68, 69]. This may have correlated with the reported seasonal variation in O157 shedding by cattle as these samples were collected in winter when cattle shed O157 in very low numbers [39, 70].

Colony PCR revealed that all the non-O157 STEC isolates carried the *eae* and *hlyA* genes (Table 2). The serogroups O103 and O26 did not match the virulence gene profiles of the respective control strains. The O121 isolate from animal #6529 matched one of the control O121 strains lacking all four virulence genes; however, the O121 antigen gene could not be amplified from this isolate, suggesting possible mutations within this gene (Table 2). Considering that the primers successfully amplified the corresponding regions from the control strains suggests that we mostly isolated variant serogroups.

PCR using DNA extracted from feces (‘fecal-DNA PCR') matched the serogroup results of colony PCR for most isolates and amplified serogroup antigens from additional samples (Table 2). Serogroups matched between the two PCR methods for isolates from animals 5620, 5657, 5760, 5825, 5916, 5935, 5938, 5982, and 6017; however, the results did not match for animal 6529 (Table 2, Supplementary Figure 1). The O103-antigen gene was also amplified from fecal DNA samples of 12 additional animals including 5776, 5787, 5845, 5868, 5901, 5915, 5985, 6069, 6120, 6171, 6542, and 6582 (Table 2). Thus, going by fecal DNA PCR alone, 21/59 (36%) animals could be considered as positive for non-O157 STEC. Virulence genes were also amplified in various combinations, from all fecal DNA extracts (Table 2), which could suggest that 100% of the animals were colonized with non-O157 STEC. However, fecal-DNA PCR results did not always correspond with the isolation of viable non-O157 STEC and hence may reflect the presence of genetic material left over from recent colonization or the presence of other STEC besides the seven adulterant serogroups targeted by our assays. Thus, results based solely on fecal DNA PCR need to be interpreted in the context of other tests and not independently.

Greater numbers of virulence genes were observed using fecal-DNA PCR in our study (Table 2, Supplementary Figure 1). This may be due to the presence of free Stx-converting bacteriophages or other STEC DNA in the absence of viable bacteria (false positives) as previously reported [71, 72]. Virulence profiles within serogroups have been recorded as being highly variable in field samples, over time, and between locations [73–75]. Hence, our observed variation in virulence profiles of STEC isolated compared to the control strains is not novel. We also observed a higher incidence of O103 by fecal DNA PCR than by culture, 36% versus 15%; O26 was isolated from 2 animal samples and O121 from 1 animal sample by culture only (Table 2). Such discrepancies between PCR and culture observed in other studies and again may be indicative of relatively older colonization versus ongoing infections with viable adulterant STEC that are more likely to contaminate the environment, hides, and hence the carcass at slaughter [46, 74, 76].

Vero cell cytotoxicity assay was used to evaluate the presence of functional toxins in the fecal samples (47, 48). Purified Shiga toxins, used as controls, demonstrated cytotoxicity on Vero cells at 1 : 64 dilution for Stx1 and 1 : 256 dilution for Stx2, which was neutralized with the corresponding antisera (Supplementary Figure 2) thus validating the test. Similar cytopathic effects (CPE) were observed with fecal extracts from 58/59 animals in Vero cell cytotoxicity assays, of which 95% (55/58) was neutralized with polyclonal anti-Stx1 and/or anti-Stx2 antisera (Table 2, Supplementary Figure 3). This Stx-related CPE could be associated with isolation of STEC and/or amplification of toxin genes via fecal DNA PCR; other STEC serogroups not targeted in our study may have also contributed to the presence of Stx in the fecal extracts (Table 2). Interestingly, CPE caused by fecal extracts from animal #6122 was neutralized with both anti-Stx1 and anti-Stx2 antisera in the absence of viable STEC or amplification of Stx genes suggesting remnant toxins from a relatively older infection with no current trace of viable STEC or DNA (Table 2). In contrast, no CPE was observed with fecal extracts from animal #5915, although the *stx2* gene was amplified from the same sample (Table 2, Supplementary Figure 2), indicating a possibly nonfunctional gene. Additionally, non-Stx factors in the fecal extracts, such as viruses, may have caused CPE observed with 3/58 fecal extracts (animals #6044, #6069, and #6148) that were not neutralized with the anti-Stx antisera (Supplementary Figure 3) [77, 78]. These variations indicate that Vero cell cytotoxicity assays require verification through neutralization steps and correlation with culture/PCR results.

STEC O103 was the predominant non-O157 STEC to be isolated from the dairy cattle evaluated in this study (Table 2). This serogroup has become one of the common non-O157 STEC to be isolated from cattle in the US and globally [37, 39, 44]. For instance, thirty calves from a closed herd in Canada were found to harbor at least one of the seven major STEC serogroups with the predominant being O103 (75.8%) and O157 (70%) [73]. Analysis of composite calf feces collected from 12 dairy farms in New Zealand identified STEC O26 (33%) to be the most prevalent serogroup, followed by O45 (25%), O103 (17%), and O121(9%) [79]. STEC O103 is also being increasingly associated with outbreaks in the US; after the venison-related O103 outbreak in 2010, three recent multistate outbreaks were associated with O103 contaminated ground beef, ground bison meat, and clover sprouts following investigations by the Centers for Disease Control and Prevention and USDA-FSIS [80–83]. This makes our observation epidemiologically relevant as well.

## 4. Conclusions

In summary, non-O157 STEC were isolated from 25% (15/59) of the animals tested using a combination of EC broth, CHROMagar STEC^TM^, and Rainbow agar O157 in this study. The two different selective agar media, used sequentially in this study, enabled differentiation of STEC from background flora and into tentative serogroups based on colony color and fluorescence phenotype. Serology and/or colony PCR was subsequently used to confirm the serogroup of the tentative non-O157 STEC as one of the “Big 6” STEC adulterants in 10/15 animals. The predominant viable non-O157 STEC serogroup isolated was O103. PCR using DNA extracted from feces verified most of the colony PCR results but also identified additional virulence and O-antigen genes from samples with no correlating culture results. Similarly, Stx-related CPE on Vero cells with fecal extracts from 55/59 animals could only be associated with the Stx gene profiles obtained with fecal DNA PCR and not culture results. Differences between culture versus fecal DNA PCR and cytotoxicity assay results suggest that the latter two assays, while alluding to the presence of STEC, may not always reflect an ongoing, viable infection with the seven adulterant STECs. Hence, this study validates that a combination of fecal culture methods are needed to distinctly isolate viable “Big 6” non-O157 STEC that pose a food safety threat. Culture methods cannot be substituted with fecal PCR or cytotoxicity assays alone which at the most could be used as primary screens to identify samples likely to harbor STEC.

## Figures and Tables

**Table 1:** Primers used in this study.

Sequence 5′⟶3″		Amplicon size (bp)	Reference
Wzx158-O26-F	GTA TCG CTG AAA TTA GAA GCG C	158	[57]
Wzx158-O26-R	AGT TGA AAC ACC CGT AAT GGC		
Wzx72-O45-F	CGT TGT GCA TGG TGG CAT	72	
Wzx72-O45-R	TGG CCA AAC CAA CTA TGA ACT		
Wzx191-O103-F	TTG GAG CGT TAA CTG GAC CT	191	
Wzx191-O103-R	ATA TTC GCT ATA TCT TCT TGC GGC		
WbdI-O111-F	TGT TCC AGG TGG TAG GAT TCG	237	
WbdI-O111-R	TCA CGA TGT TGA TCA TCT GGG		
Wzx189-O121-F	AGG CGC TGT TTG GTC TCT TAG a	189	
Wzx189-O121-R	GAA CCG AAA TGA TGG GTG CT		
Wzx135-O145-F	AAA CTG GGA TTG GAC GTG G	135	
Wzx135-O145-R	CCC AAA ACT TCT AGG CCC G		
Stx (Stx1/2)-F1 Stx (Stx 1/2)-R1	TTT GTY ACT GTS ACA GCW GAA GCY TTA CG CCC CAG TTC ARW GTR AGR TCM ACD TC	131 bp (stx1) 128 bp (stx2)	
Eae-F	CAT TGA TCA GGA TTT TTC TGG TGA TA	102	
Eae-R1	CTC ATG CGG AAA TAG CCG TTM		
O26-F	CAATGGGCG GAAATTTTAGA	155	[56]
O26-R	ATAATTTTCTCTGCCGTCGC		
O121	TCCAACAATTGTCGTGAAA	628	
O121-R	AGAAAG TGTGAAATGCCCGT		
Stx1-F	ATAAATCGCCATTCGTTGACTAC	180	[59]
Stx1-R	AGAACGCCCACTGAGATCATC		
Stx2-F	GGCACTGTCTGAAACTGCTCC	255	
Stx2-R	TCGCCAGTTATCTGACATTCTG		
HlyA-F	GCATCATCAAGCGTACGTTCC	534	
HlyA-R	AATGAGCCAAGCTGGTTAAGCT		

^1^Degenerate nucleotide codes are as follows: Y (C, T); S (C, G); W (A, T); R (A, G); M (A, C); D (A, G, T).

**Table 2:** Assay results per animal.

Animal number	Number of mauve, fluorescent colonies shown as CFU/ml^1^	Latex agglutination−based serogroup of non−O157 isolates	Colony lysate PCR											Fecal−DNA PCR											Vero cell assay			
			O103	O111	O121	O145	O26	O45	*eae*	*stx*	*stx*1	*stx*2	*hly*A	O103	O111	O121	O145	O26	O45	*eae*	*Stx*	*stx*1	*stx*2	*hly*A	CPE dilution^3^	CPE scores	No CPE^4^ with anti-Stx1 sera	No CPE with anti-Stx2 sera
5602	−	−	−	−	−	−	−	−	−	−	−	−	−	−	−	−	−	−	−	+	+	−	+	+	8	1	n	y
5620	7 × 10^3^	O103	+	−	−	−	−	−	+	−	−	−	+	+	−	−	−	−	−	−	+	+	+	+	64	3	y	y
5657	5 × 10^4^	O103	+	−	−	−	−	−	+	−	−	−	+	+	−	−	−	−	−	+	+	+	+	+	8	1	y	y
		NT^2^	−	−	−	−	−	−	+	−	−	−	+															
5724	−	−	−	−	−	−	−	−	−	−	−	−	−	−	−	−	−	−	−	+	+	+	+	+	0	3	y	y
5743	−	−	−	−	−	−	−	−	−	−	−	−	−	−	−	−	−	−	−	+	+	+	+	+	16	1	n	y
5759	1 × 10^3^	NT	−	−	−	−	−	−	−	−	−	−	+	−	−	−	−	−	−	−	+	+	+	+	2	2	y	y
5760	1 × 10^8^	O103	+	−	−	−	−	−	+	−	−	−	+	+	−	−	−	−	−	+	+	+	+	+	4	1	n	y
5771	4 × 10^4^	NT	−	−	−	−	−	−	+	+	+	+	+	−	−	−	−	−	−	+	+	+	+	+	2	1	y	y
5776	5 × 10^4^	NT	−	−	−	−	−	−	+	+	+	−	+	+	−	−	−	−	−	−	+	+	+	+	4	1	y	y
5787	−	−	−	−	−	−	−	−	−	−	−	−	−	+	−	−	−	−	−	+	+	+	+	+	4	1	y	n
5800	−	−	−	−	−	−	−	−	−	−	−	−	−	−	−	−	−	−	−	−	+	+	+	+	8	1	y	y
5825	4 × 10^4^	O103	+	−	−	−	−	−	+	−	−	−	+	+	−	−	−	−	−	+	+	−	+	+	4	1	n	y
5845	−	−	−	−	−	−	−	−	−	−	−	−	−	+	−	−	−	−	−	+	+	−	+	+	2	2	n	y
5868	−	−	−	−	−	−	−	−	−	−	−	−	−	+	−	−	−	−	−	+	+	+	+	+	4	2	y	y
5874	−	−	−	−	−	−	−	−	−	−	−	−	−	−	−	−	−	−	−	+	+	+	+	+	8	1	y	y
5875	−	−	−	−	−	−	−	−	−	−	−	−	−	−	−	−	−	−	−	+	+	+	+	+	2	1	y	y
5889	−	−	−	−	−	−	−	−	−	−	−	−	−	−	−	−	−	−	−	+	+	+	+	+	4	1	y	y
5901	−	−	−	−	−	−	−	−	−	−	−	−	−	+	−	−	−	−	−	+	+	+	+	+	8	1	y	y
5915	−	−	−	−	−	−	−	−	−	−	−	−	−	+	−	−	−	−	−	+	+	−	+	+	0	0	n/a	n/a
5916	4 × 10^4^	O103	+	−	−	−	−	−	+	−	−	−	+	+	−	−	−	−	−	+	+	+	+	+	4	2	y	y
5922	−	−	−	−	−	−	−	−	−	−	−	−	−	−	−	−	−	−	−	−	+	+	+	+	2	3	y	y
5935	2.4 × 10^4^	O103	+	−	−	−	−	−	+	−	−	−	+	+	−	−	−	−	−	−	+	+	+	+	0	3	y	y
		O26	−	−	−	−	+	−	+	−	−	−	+															
5938	6.5 × 10^6^	O103	+	−	−	−	−	−	+	−	−	−	+	+	−	−	−	−	−	+	+	+	+	+	2	1	y	y
5939	−	−	−	−	−	−	−	−	−	−	−	−	−	−	−	−	−	−	−	+	+	+	+	+	2	2	y	y
5949	−	−	−	−	−	−	−	−	−	−	−	−	−	−	−	−	−	−	−	−	+	+	+	+	8	1	y	y
5950	−	−	−	−	−	−	−	−	−	−	−	−	−	−	−	−	−	−	−	+	−	+	+	+	8	3	y	n
5964	−	−	−	−	−	−	−	−	−	−	−	−	−	−	−	−	−	−	−	+	+	+	+	+	8	3	n	y
5966	−	−	−	−	−	−	−	−	−	−	−	−	−	−	−	−	−	−	−	+	+	+	+	+	8	1	y	y
5982	3.5 × 10^8^	O103	+	−	−	−	−	−	+	−	−	−	+	+	−	−	−	−	−	+	+	−	+	+	0	2	n	y
5985	3 × 10^8^	NT	−	−	−	−	−	−	+	−	−	−	+	+	−	−	−	−	−	+	+	+	+	+	8	1	y	y
5996	−	−	−	−	−	−	−	−	−	−	−	−	−	−	−	−	−	−	−	+	+	+	+	+	4	1	y	y
6009	−	−	−	−	−	−	−	−	−	−	−	−	−	−	−	−	−	−	−	+	+	+	+	+	4	3	y	n
6011	−	−	−	−	−	−	−	−	−	−	−	−	−	−	−	−	−	−	−	+	+	+	+	+	8	1	y	y
6012	−	−	−	−	−	−	−	−	−	−	−	−	−	−	−	−	−	−	−	+	+	+	+	+	2	1	y	y
6013	−	−	−	−	−	−	−	−	−	−	−	−	−	−	−	−	−	−	−	−	+	+	+	+	0	2	y	y
6017	2 × 10^7^	O103	+	−	−	−	−	−	+	−	−	−	+	+	−	−	−	−	−	+	+	+	+	+	2	1	y	y
6044	−	−	−	−	−	−	−	−	−	−	−	−	−	−	−	−	−	−	−	+	+	+	+	+	2	1	n	n
6049	−	−	−	−	−	−	−	−	−	−	−	−	−	−	−	−	−	−	−	+	+	+	+	+	0	3	y	y
6069	−	−	−	−	−	−	−	−	−	−	−	−	−	+	−	−	−	−	−	+	+	+	+	+	2	1	n	n
6082	−	−	−	−	−	−	−	−	−	−	−	−	−	−	−	−	−	−	−	−	+	+	+	+	2	3	y	n
6089	−	−	−	−	−	−	−	−	−	−	−	−	−	−	−	−	−	−	−	+	+	+	+	+	2	4	y	n
6101	−	−	−	−	−	−	−	−	−	−	−	−	−	−	−	−	−	−	−	+	+	+	+	+	2	3	y	n
6120	−	−	−	−	−	−	−	−	−	−	−	−	−	+	−	−	−	−	−	+	+	+	+	+	2	1	y	y
6122	−	−	−	−	−	−	−	−	−	−	−	−	−	−	−	−	−	−	−	+	−	−	−	+	2	2	y	y
6130	−	−	−	−	−	−	−	−	−	−	−	−	−	−	−	−	−	−	−	+	+	+	+	+	2	3	y	y
6137	−	−	−	−	−	−	−	−	−	−	−	−	−	−	−	−	−	−	−	−	+	+	+	−	4	2	y	y
6141	−	−	−	−	−	−	−	−	−	−	−	−	−	−	−	−	−	−	−	−	+	+	+	+	2	2	n	y
6148	2 × 10^5^	NT	−	−	−	−	−	−	+	−	−	−	+	−	−	−	−	−	−	+	+	+	+	+	2	3	n	n
6150	−	−	−	−	−	−	−	−	−	−	−	−	−	−	−	−	−	−	−	+	+	+	+	+	2	2	n	y
6153	−	−	−	−	−	−	−	−	−	−	−	−	−	−	−	−	−	−	−	−	+	+	+	−	0	1	n	y
6171	−	−	−	−	−	−	−	−	−	−	−	−	−	+	−	−	−	−	−	+	−	+	+	+	0	3	y	y
6177	−	−	−	−	−	−	−	−	−	−	−	−	−	−	−	−	−	−	−	−	+	+	+	+	4	3	y	y
6179	−	−	−	−	−	−	−	−	−	−	−	−	−	−	−	−	−	−	−	−	+	+	+	−	4	3	y	y
6507	−	−	−	−	−	−	−	−	−	−	−	−	−	−	−	−	−	−	−	−	+	+	+	+	8	1	n	y
6509	−	−	−	−	−	−	−	−	−	−	−	−	−	−	−	−	−	−	−	−	+	+	+	+	2	4	y	y
6529	5 × 10^4^	O26	−	−	−	−	−	−	+	−	−	−	+	−	−	−	−	−	−	−	+	+	+	+	4	3	n	y
		O121	−	−	−	−	−	−	−	−	−	−	−															
6542	−	−	−	−	−	−	−	−	−	−	−	−	−	+	−	−	−	−	−	−	+	+	+	+	2	1	y	y
6561	−	−	−	−	−	−	−	−	−	−	−	−	−	−	−	−	−	−	−	+	+	+	+	+	2	1	n	y
6582	−	−	−	−	−	−	−	−	−	−	−	−	−	+	−	−	−	−	−	+	+	+	+	+	0	3	y	y

^1^Putative non-O157 STEC forming mauve, fluorescent colonies on CHROMagar STEC^TM^, and postenrichment in EC broth were further analyzed by serology and colony PCR. ^2^NT, not typed to either of the “Big 6” non-O157 STEC serogroups by latex agglutination. ^3^The last dilution at which CPE still observed on the Vero cells was selected when setting up the toxin neutralization assays. ^4^y, yes; n, no; n/a, not applicable as no CPE was observed when at undiluted concentrations.

## Data Availability

No data were used to support the findings of this study.

## Supplementary Materials

Supplementary Materials Supplementary Figure 1. Representative colony (a) and fecal DNA (b) PCR results are shown for animal 5760. PCR reactions were analyzed by electrophoresis on a 4% agarose gel and loaded in lanes 1–13 as 100 bp ladder, O103, O111, O121, O145, O26, O45, eae, stx, stx1, stx2, hlyA, and 2-log ladder. Amplicon sizes in bp, as expected, are shown. Supplementary Figure 2. Vero cell cytotoxicity assay. I, assay controls with media only (a) and media and antisera (b) on Vero cells are shown. II, diluted Stx and diluted Stx with anti-Stx sera are shown as described in the inserted legends. Cytopathic effects of Stx1 at 1 : 64 dilution (c) and Stx2 at 1 : 256 dilution (e) on the Vero cells and also the protection of the Vero cells in the presence of antisera, anti-Stx1 (d) and anti-Stx2 (f), are shown. III, absence of cytopathic effects with undiluted fecal extracts from animal 5951 is shown (g, h). All images were captured at 10x magnification using an inverted microscope. Supplementary Figure 3. Vero cell cytotoxicity assay with fecal extracts. Examples of various effects of fecal extracts on Vero cells are shown as described in the inserted legends. Images were captured at 10x magnification using an inverted microscope.
