# Supplementary material for: Evaluation of Cattle for Naturally Colonized Shiga Toxin-Producing Escherichia coli Requires Combinatorial Strategies
Source: Int J Microbiol. 2021 Apr 1;2021:6673202. doi: 10.1155/2021/6673202 (PMC8032530; doi:10.1155/2021/6673202)
Supplement: Supplementary Materials — Supplementary Figure 1. Representative colony (a) and fecal DNA (b) PCR results are shown for animal 5760. PCR reactions were analyzed by electrophoresis on a 4% agarose gel and loaded in lanes 1–13 as 100 bp ladder, O103, O111, O121, O145, O26, O45, eae, stx, stx1, stx2, hlyA, and 2-log ladder. Amplicon sizes in bp, as expected, are shown. Supplementary Figure 2. Vero cell cytotoxicity assay. I, assay controls with media only (a) and media and antisera (b) on Vero cells are shown. II, diluted Stx and diluted Stx with anti-Stx sera are shown as described in the inserted legends. Cytopathic effects of Stx1 at 1 : 64 dilution (c) and Stx2 at 1 : 256 dilution (e) on the Vero cells and also the protection of the Vero cells in the presence of antisera, anti-Stx1 (d) and anti-Stx2 (f), are shown. III, absence of cytopathic effects with undiluted fecal extracts from animal 5951 is shown (g, h). All images were captured at 10x magnification using an inverted microscope. Supplementary Figure 3. Vero cell cytotoxicity assay with fecal extracts. Examples of various effects of fecal extracts on Vero cells are shown as described in the inserted legends. Images were captured at 10x magnification using an inverted microscope. [file 6673202.f1.zip › 6673202.f1/SuppFigure-3-IKudva.pdf]

A B C

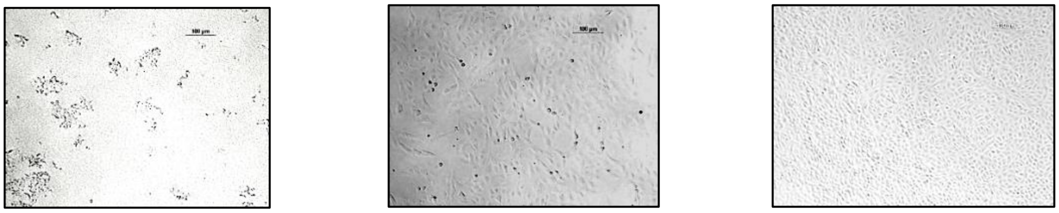

A. Vero cells with 5620 fecal extract at 1:64 showing CPE
B. Vero cells with 5620 fecal extract at 1:64 + Anti-Stx1 antisera showing protection
C. Vero cells with 5620 fecal extract at 1:64 + Anti-Stx2 antisera showing protection

D E F

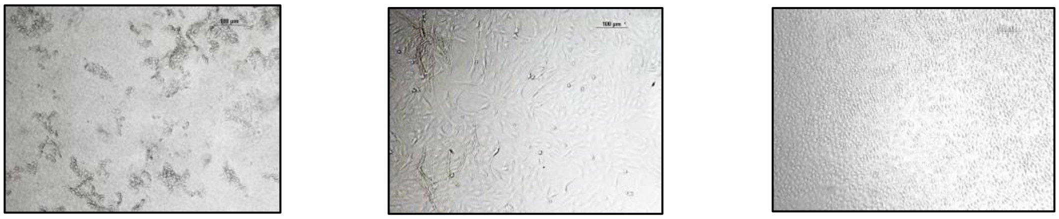

D. Vero cells with 5787 fecal extract at 1:4 showing CPE
E. Vero cells with 5787 fecal extract at 1:4 + Anti-Stx1 antisera showing protection
F. Vero cells with 5787 fecal extract at 1:4 + Anti-Stx2 antisera showing no protection

G H I

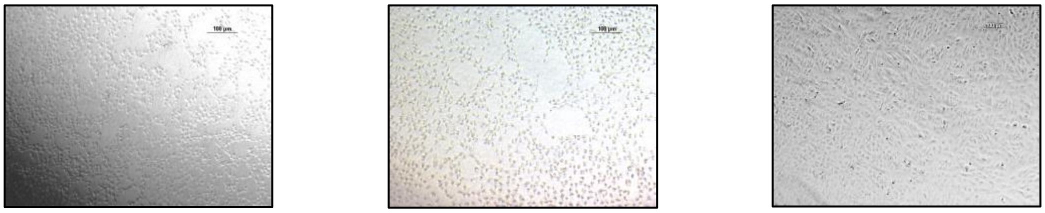

G. Vero cells with 6507 fecal extract at 1:8 showing CPE
H. Vero cells with 6507 fecal extract at 1:8 + Anti-Stx1 antisera showing no protection
I. Vero cells with 6507 fecal extract at 1:8 + Anti-Stx2 antisera showing protection

J K L

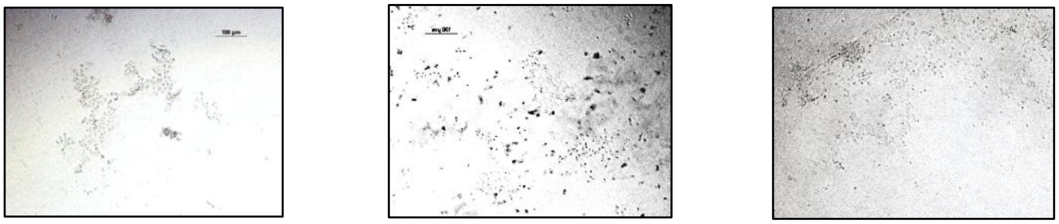

J. Vero cells with 6148 fecal extract at 1:2 showing CPE
K. Vero cells with 6148 fecal extract at 1:2 + Anti-Stx1 antisera showing no protection
L. Vero cells with 6148 fecal extract at 1:2 + Anti-Stx2 antisera showing no protection

Supplementary Figure 3. Vero cell cytotoxicity assay with fecal extracts. Examples of various effects of fecal extracts on Vero cells is shown as described in the inserted legends. Images were captured at 10x magnification using an inverted microscope.
